# COVID-19 infection and treatment-resistant cocaine-induced pyoderma gangrenosum: A case report

**DOI:** 10.1016/j.amsu.2022.103828

**Published:** 2022-05-18

**Authors:** Jennifer Adams, Daniel Habenicht, Yaman Gibran

**Affiliations:** a University of Texas Rio Grande Valley - School of Medicine, 1201 W University Dr, Edinburg, TX, USA; b Department of Internal Medicine, University of Texas Rio Grande Valley, Valley Baptist Medical Center, Harlingen, TX, USA

**Keywords:** Pyoderma gangrenosum, COVID-19, Cocaine-induced

## Abstract

**Introduction:**

Pyoderma gangrenosum (PG) is a rare neutrophilic dermatosis of non-infectious etiology. Cocaine-induced PG (CIPG) is a documented clinical variant.

**Case presentation:**

We report an exceptional case of cocaine-induced PG flare unresponsive to conventional treatment in the context of positive COVID status. A 41year-old male with past medical history of recent COVID infection, pyoderma gangrenosum and chronic cocaine abuse presented with acutely worsening multifocal ulcerations covering multiple limbs approximately 30% body surface area (BSA) one day after cocaine use. After hospitalization for ten days with no improvement in cutaneous symptoms, he was transferred to a burn center for disease control with biologics.

**Discussion:**

The previous temporal relationship between disease outbreak and cocaine consumption and improvement after its discontinuation no longer remained in the setting of COVID positive status. This is the first case in literature of extensive and treatment-refractory PG in a COVID-positive patient with recent cocaine use.

**Conclusion:**

This case highlights the importance of further investigation on the connection between COVID infection and PG and the need for establishing treatment guidelines for PG.

## Highlights

• This is the first recorded case of treatment-refractory cocaine-induced pyoderma gangrenosum with active COVID infection.
• Further studies on the relationship between active COVID infection and Pyoderma Gangrenosum exacerbations are warranted.
• Multidisciplinary care is needed in tailoring management to persistent pyoderma gangrenosum in patients with COVID infection.

## 1 Introduction

Pyoderma gangrenosum (PG) is an ulcerative skin disorder characterized histologically by the accumulation of neutrophils in the dermis. It clinically manifests as one or more painful, purulent ulcers with undermined borders on sites of normal or traumatized skin. About 50% of cases are associated with underlying systemic autoimmune conditions and malignancy [1]. A few cases are reported to be induced by cocaine use (CIPG). The clinical presentation of CIPG is often more widespread than the classic forms, with higher in quantity of lesions, which are larger in diameter and located predominantly on the trunk. Pre-auricular lesions are noted to be associated with CIPG [3]. The interval between the cocaine consumption and the onset of symptoms is generally 7 days [2]. The symptoms typically relapse and are responsive to treatments with the cessation of cocaine use. This case is the first reported case of cocaine-induced PG flare unresponsive to conventional treatment in the context of positive COVID status.

This work has been reported in line with the SCARE 2020 criteria [11].

## 2 Case Presentation

**Fig. 1:**
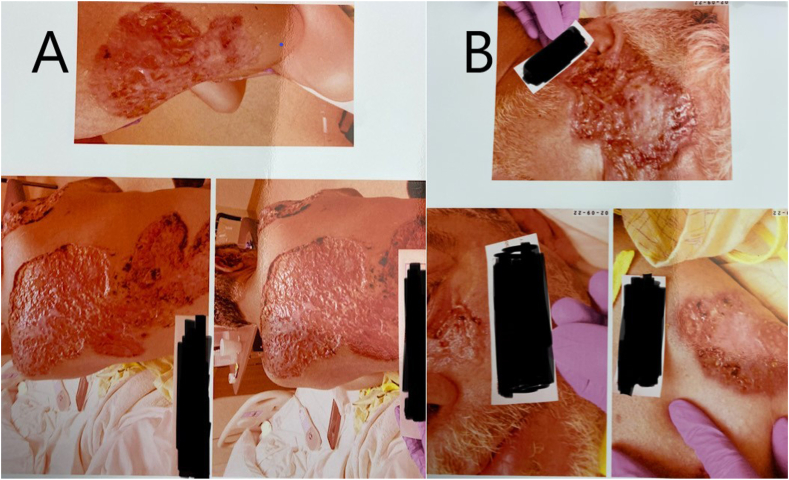
A. Necrotic ulcerated lesion covering greater than 60% of the entire back beginning at the right and left shoulders. The borders are sharply defined and irregular; the area is raw with thick brown crusting near the flank. B. New ulcerations at the right temporal area of the face extending to the ear and inferior to the left eye.

A 41 year-old male with past medical history of pyoderma gangrenosum, type 2 diabetes mellites, hypertension, and cocaine abuse presented to the emergency department with worsening wound pain and ulceration involving upper back, left trunk, right arm (Fig. 1A), left shoulder and Left infraorbital maxilla and right temporal area extending behind the ear (Fig. 1B). The patient has a 20-year history of cocaine use. Family history was noncontributory. Psychosocial history is significant for depression. The patient report that he does not use medications. The physical exam revealed extensive cribriform ulcerations with irregular borders and extensive purulent drainage (Fig. 1). Ulceration spanned approximately 30% of body surface area. The patient reported that the pain began to gradually worsen about 8 days before arrival to hospital. He denied fever, cough, sore throat, chest pain, shortness of breath, nausea, vomiting, and diarrhea. Patient has an established diagnosis of pyoderma gangrenosum and has been admitted in the past for the same complaints. He reported use of cocaine 10 days prior to admission after which his symptoms worsened. Patient tested positive for Covid-19. The results of a laboratory work-up were normal, including a complete blood count, general biochemistry tests, an autoimmune profile, an antinuclear antibody panel, a lupus anticoagulant, cryoglobulin, C-reactive protein test, complement levels and erythrocyte sedimentation rate. The patient tested negative for hepatitis B, hepatitis C and HIV. A spirochetes stain, C-ANCA, p-ANCA testing was negative. Biopsy of an ulcer done on a previous admission one month prior showed perivascular plasmacytosis with focal vasculitis, suggestive of PG. Wound culture grew Klebsiella pneumonia ESBL, Pseudomonas, Morganella Morgagni and coagulase-negative staph. The patient's hospital care was managed by the on-call hospitalist and the residents of the hospital's internal medicine program.

Based on the sensitivity testing, the patient was administered Vancomycin 15 mg/kg intravenously every 12 hours and Meropenem 500 mg every 8 hours. The PG was treated with IV methylprednisolone and topical clobetasol dipropionate 0.05% ointment, which is the recommended treatment for immune-related skin manifestations. The patient's ulceration flares did not remit with in-hospital therapy. Given his history of multiple readmissions with recurrence of similar clinical picture and admitted to cocaine relapse after discharges. Because of the severity of the condition and the extensiveness of the area affected, the recommendation was by the hospitalist and the plastic surgeon to refer the patient to a burn center. The consulting dermatologist outlined the possibility of starting a regimen of prednisone 50 mg daily and mycophenolate mofetil 1000 mg twice a day and eventual transition to infliximab for long-term treatment in addition to continued counseling regarding his cocaine use.

## 3 Discussion

Differential diagnoses included ANCA-associated and leukocytoclastic vasculitis. This case is the first reported case of cocaine-induced PG flare unresponsive to conventional treatment in the context of positive COVID status.astic vasculitis. A recent review of 20 cases of CIPG showed that 73% of the cases were positive for p-ANCA and 43% had at least one antiphospholipid antibody (APL), most frequently the anticardiolipin IgM [1]. Few other cases with negative ANCA and APL, similar to our patient, have also described [1,[3], [4], [5]]. Our patient had been diagnosed with pyoderma gangrenosum in December 2019 and has been admitted in the past for the same complaints and negative findings of other systemic disease, c-ANCA, p-ANCA, hepatitis, hematologic disorders.

One previous case reported a middle-aged woman with an exacerbation of PG after cocaine use refractory to treatment with IV corticosteroids and cyclosporine [4]. Disease control was eventually achieved with oral corticosteroids together with mycophenolic acid, infliximab, and abstinence from cocaine consumption. Another case describes a 46-year-old man with a 1-year history of generalized PG resistant to treatments [6]. He presented with 22 cribriform ulcers and atrophic scars located predominantly on the trunk, with two lesions in the pre-auricular region previously treated with prednisone 50 mg daily combined with cyclosporine but had multiple disease relapses. In these cases, the temporal relationship between disease flare and improvement after cocaine-use discontinuation raises the possibility of cocaine as both an etiology of PG as well as etiology for therapy resistance. In our case, this temporal relationship was not present during this hospitalization and ulcer progression did not respond to therapy or cessation of drug use.

Levamisole-induced vasculitis was also considered in the differential. Levamisole is a common adulterant added to cocaine [7]. Unfortunately, levamisole was not included in the initial urine drug screen, and the search for levamisole after 48 hours of hospitalization is often negative considering its short half-life of 5.6 h [8]. It is therefore difficult to confirm its presence in cases of CIPG.

A final consideration is the possibility that the PG flare or its refractoriness to treatment may be a cutaneous manifestation of COVID-19. The mechanisms behind cutaneous manifestations in COVID-19 are still under investigation, but likely involve the indirect effects of immune system hyperactivity and hypercoagulability. Immunological similarities between the pathogenesis of COVID-19 and PG include the significant roles of proinflammatory cytokines and neutrophilic abnormalities [9]. One recent case documented a possible relationship between PG and COVID. Ten days after testing positive for COVID-19 PCR, a 71-year-old man developed painful and pruritic pustules on his left scrotum that quickly ulcerated within a few days and progressed to the penis, groin, buttocks, and abdomen over a span of three months [10]. The reported case highlight the possibility that the COVID-19 spike protein may serve as a possible PG immune trigger. PG is associated with an increase in proinflammatory cytokines, including interleukin-12 (IL-12), IL-23, tumor necrosis factor (TNF)-alpha, and IL-6 ^9^, which are also involved in the pathogenesis of COVID-19. Biologic agents targeting TNF-α, IL-12, and IL-23 have been effective in treating PG [10]. The National Institutes of Health (NIH) COVID-19 treatment guidelines panel recommends tocilizumab, an IL-6 antagonist, along with systemic corticosteroids for rapidly deteriorating COVID-19 patients and suggests through one case study that the drug may be effective for PG treatment [12]. Future studies investigating the similarities in both the inflammatory cytokines and signaling pathways involved in the two conditions may inform better-tailored management strategies for affected patients.

The therapeutical approach to CIPG is complicated. In addition to wound care, systemic therapy such as corticosteroids, immunosuppressive and biological therapy may be needed to achieve remission in cases of extensive PG, however cessation of cocaine use is imperative. Recurrences are almost always linked to a new exposure, such as drug use or infection. Patient declined to provide statement of perspective.

## 4 Conclusion

We present a rare case of CIPG, previously controlled with systemic steroids, presenting with a worsening and treatment-resistant flare in the context of COVID-19 infection and recent cocaine use. This case emphasizes the necessity of studies to elucidate the immunopathological connection between COVID and treatment-resistant PG. In addition to drug abstinence, which is paramount to optimizing treatment response and preventing flares, the treatment of PG remains a challenge. The prognosis of CIPG remains unpredictable.

## Patient perspective

N/A.

## Funding

This research did not receive any specific grant from funding agencies in the public, commercial, or not-for-profit sectors.

## Informed consent

Written informed consent was obtained from the patient for publication of this case report and accompanying images. A copy of the written consent is available for review by the Editor-in-Chief of this journal on request.

## Provenance and peer review

Not commissioned, externally peer-reviewed.

## Consent

Obtained.

## Ethical approval

N/A.

## Sources of funding

None.

## Author contribution

JA was involved in the writing of the manuscript. Y and DH were involved in the editing and supervision of the manuscript.

## Research registration (for case reports detailing a new surgical technique or new equipment/technology)

1. Name of the registry: N/A
2. Unique identifying number or registration ID: N/A
3. Hyperlink to your specific registration (must be publicly accessible and will be checked): N/A

## Guarantor

Jose Campo-Maldonado MD.

## Declaration of competing interest

All authors declare no conflicts of interest.
